# Identification and analgesic activity study of analgesic protein Ⅶ-2 from *Naja naja atra* venom

**DOI:** 10.1590/1678-9199-JVATITD-2023-0099

**Published:** 2024-09-02

**Authors:** Yao Sun, Gen-Bao Zhang, Shu Li, Xiao-Yu Liu, Lei Chen, Peng-Ju Bao

**Affiliations:** 1 Department of Pathophysiology, Institute of Snake Venom, Wannan Medical College, Wuhu, China.; 2 School of Anesthesiology, Wannan Medical College, Wuhu, China.; 3 Department of Physiology, Institute of Snake Venom, Wannan Medical College, Wuhu, China.

**Keywords:** Naja naja atra, Venom, Acid-sensing ion channel 1a, Capillary electrophoresis

## Abstract

**Background:**

Acid-sensing ion channel 1a (ASIC1a) plays a critical role in physiological and pathological processes. To further elucidate the biological functions of ASICs and their relationships with disease occurrence and development, it is advantageous to investigate and develop additional regulatory factors for ASICs.

**Methods:**

In this study, cation exchange chromatography was used to separate seven chromatographic components from *Naja naja atra* venom. Capillary electrophoresis was employed to detect that Ⅶ peak component containing a main protein Ⅶ-2, which could bind to ASIC1a. The analgesic effects of Ⅶ-2 protein were determined using hot plate methods, and ASIC1a expression in spinal cord tissue from rats with inflammatory pain was detected using western blot.

**Results:**

The purified Ⅶ-2 protein named *Naja naja atra* venom-Ⅶ-2 (NNAV-Ⅶ-2) was obtained by Sephadex G-50 gel filtration, which exhibited a single band on sodium dodecyl sulfate-polyacrylamide gel electrophoresis with a molecular weight of 6.7 kD. Remarkably, the NNAV-Ⅶ-2 protein demonstrated a significant analgesic effect and downregulated ASIC1a expression in the spinal cord tissue of rats with inflammatory pain.

**Conclusions:**

The analgesic mechanism of the NNAV-Ⅶ-2 protein may be associated with its binding to ASIC1a, consequently downregulating ASIC1a expression in neural tissues.

## Background

Acid-sensing ion channels (ASICs), belonging to the degenerin/epithelial sodium channel family, constitute a class of cationic protein complexes present on the cell membrane. They are widely expressed in the central and peripheral nervous systems [1, 2]. Six ASIC subunits encoded by four genes have been identified, namely ASIC1a, ASIC1b, ASIC2a, ASIC2b, ASIC3, and ASIC4 [3]. Among these subunits, ASIC1a plays a critical role in acid damage. Studies [4] have demonstrated that ASIC1a is involved in the process of cerebral ischemia and plays a role in exacerbating cell damage. Conversely, ASIC1a knockout mice exhibit remarkable tolerance to nerve damage induced by cerebral ischemia, resulting in a substantial 60% reduction in infarct volume. Inhibiting the ASIC1a channel also confers considerable tolerance to damage. Furthermore, investigations [5] have reported a significant increase in ASIC1a expression in the spinal dorsal horn of mice in a formalin-induced inflammatory pain model. Inhibiting the ASIC1a channel can mitigate central allergies, exhibiting anti-nociceptive effects [6, 7]. Current research efforts are increasingly dedicated to investigating the biological functions of ASICs and their relationships with disease occurrence and development. Exploring and developing more regulators that can target ASICs is a crucial breakthrough in addressing this challenge.

Recently, ligands that regulate ASIC1a function have been discovered in animal peptides [8, 9]*.* For instance, Psalmotoxin-1 (PcTx-1) [10, 11], a peptide derived from tarantula venom, has been shown to activate the endogenous enkephalin pathway by blocking ASIC1a, demonstrating strong analgesic properties that effectively inhibit rodent thermal, mechanical, chemical, inflammatory, and neuropathic pain. Additionally, mambalgins [12-14]*,* toxins polypeptides obtained from African mamba venom, exert analgesic effects by inhibiting ASIC1 channel activation in both central and peripheral nerves. Capillary electrophoresis [15, 16] was employed in this study to identify a protein from *Naja naja atra* venom that interacts with ASIC1a. This protein exhibits a significant analgesic effect and markedly enhances the downregulation of ASIC1a in the nerve tissue of rats experiencing inflammatory pain. These findings offer a crucial theoretical foundation for further investigating the analgesic mechanisms of toxins and the functions of ASICs.

## Methods

### Ethical statement

B&K Universal Group Limited (Shanghai, China; license number, 2018-0006) provided mice and rats for this study. Before the start of the experiments, all staff and investigators received training in the humane handling of animals. The Animal Welfare and Ethics Committee of Wannan Medical College granted approval for all animal-related experiments (LLSC-2021-197).

### Materials and animals

Crude *Naja naja atra* venom was purchased from the Qimen Institute of Snake Venom (Huangshan, China). CM Sephadex C-25 was procured from Pharmacia (Stockholm, Sweden). Rat ASIC1a ELISA Kit was obtained from Jiangsu Enzyme-Free Industrial Co., Ltd (Jiangsu, China). Anti-rat ASIC1a antibody was purchased from HUABIO (Hangzhou, China). The sodium dodecyl sulfate-polyacrylamide gel electrophoresis (SDS-PAGE) low molecular mass standard protein marker was procured from Thermo Scientific (Massachusetts, America). Membrane Protein and Cytoplasmic Protein Extraction Kits were obtained from Sangon Biotech (Shanghai, China). PcTx-1 was purchased from MedChemExpress (New Jersey, America).

This study included 30 SPF-grade adult female mice weighing 18-22 g and 40 SPF-grade male Sprague Dawley (SD) rats weighing 240-260 g. These animals were housed in well-ventilated cages, had free access to food and water, and were maintained at a temperature of 20 ℃-25 ℃ with standard lighting conditions.

###  Isolation of snake venom from *Naja naja atra*

We dissolved 0.5 g of crude venom powder in 5 mL of 0.01 mol/L sodium phosphate buffer (pH 6.0). The mixture was centrifuged at 10,000 × *g* for 15 minutes at 4℃. Subsequently, we filtered the supernatant using a 0.22 μm filter membrane and loaded it onto a CM Sephadex C-25 cation exchange chromatography column (1.6 × 50 cm) equilibrated with 0.01 mol/L phosphate buffer (pH 6.0). Elution was performed with the same sodium phosphate buffer plus 0.5 mol/L NaCl (400mL of each of two solutions) as a gradient with a flow rate of 0.8 mL/min at room temperature and monitored at 280 nm. These procedures were performed using an AKTA Purifier. The resulting fractions were collected and stored in sealed bottles at −80℃ until needed after desalination and dehydration. 

### Capillary electrophoresis to analyze venom protein interacting with ASIC1a

*Preparation of an inflammatory model induced by formalin*

We injected 100 μL of 2.5% formalin solution subcutaneously into the plantar surface of the rat's hind paw [17]. We then observed and recorded injury behavior (licking and lifting the hind palm off the bottom of the box) after the injection to verify the effect of model replication.

*Extraction of membrane proteins from rat spinal cord tissue*

Rats were anesthetized by intraperitoneally injecting 1.5% pentobarbital sodium (2 mL/kg) six hours after formalin injection. Then, 75% ethanol was used to disinfect the skin, and the spinal cord tissue was dissected [18]. An appropriate amount of precooled PBS was added to the tissue, which was centrifuged at 3,000 × *g* for three minutes at 4 ℃. The supernatant was discarded, and the tissue was washed twice with PBS. Next, 1 mL of precooled membrane protein extract buffer A was added to the tissue, and the homogenate was prepared by crunching with a pestle. The homogenate was centrifuged at 1,000 × *g* for 10 minutes at 4 ℃. The supernatant was transferred to a precooled centrifuge tube and centrifuged at 12,000 × *g* for 60 minutes at 4 ℃. The supernatant was discarded, further, 500 μL of precooled buffer B was added to the pellet. After oscillating for 10 seconds in a vortex and placing it on ice for 30 minutes, the mixture was centrifuged at 12,000 × *g* for 10 minutes at 4℃. The supernatant contained membrane proteins and was stored at −80℃ until further use.

*Electrophoresis conditions*

The quartz capillary measures 48.5 cm in length (with an effective length of 40 cm) and has an internal diameter of 50 μm (Beckman Coulter, USA). The separation voltage was 13 kV, with a detection wavelength of 198 nm, and 50 mmol/L borate buffer (pH 12.0) as the running buffer. The operating temperature was set at 20 ℃, and the pressure injection was conducted at 3447.38 Pa for five seconds. All procedures were performed using capillary electrophoresis instrumentation from Beckman Coulter. 

*Pretreatment of capillary*

We rinsed the new capillary with 1 mol/L sodium hydroxide, 1 mol/L hydrochloric acid, and ultrapure water for 30 minutes each. Before injection, the capillary was rinsed with 0.2 mol/L sodium hydroxide, ultrapure water, and 50 mmol/L borate buffer (pH 12.0) for three minutes each. After every 6 consecutive injections, the buffer solution was replaced. All solutions were filtered through a 0.22 μm filter membrane.

*Capillary electrophoresis of Ⅶ peak samples*

The Ⅶ peak solution was diluted to concentrations of 1.2, 0.6, 0.3, and 0.15 mg/mL with ultrapure water. Subsequently, the Ⅶ peak samples were analyzed with 50 mmol/L borate buffer (pH 12.0) as the running buffer.

*Capillary electrophoresis of Ⅶ peak-treated samples*

Fifty μL of the extracted spinal tissue membrane protein sample was added to the bottom of the well of the ELISA plate containing the anti-rat ASIC1a antibody; the plate was gently shaken. After sealing, the plate was incubated at 37 ℃ for 30 minutes. Subsequently, the supernatant was discarded, and the plate was washed three times with an ELISA washing solution. Then, the plate was washed two times using ultrapure water. Next, 50 μL of Ⅶ peak solution (0.3 mg/mL) was added to the well and the solution was mixed thoroughly. The ELISA plate was incubated at 37 ℃. The supernatants were obtained as treated samples after incubation for 5, 10, or 20 minutes. The Ⅶ peak-treated samples were analyzed using capillary electrophoresis under the above electrophoresis conditions. It was discovered that the protein could bind to ASIC1a, which we named *Naja naja atra* venom-Ⅶ-2 (NNAV-Ⅶ-2).

### Further purification and the relative molecular mass determination of NNAV-Ⅶ-2 protein 

After desalination, dehydration, and determination of protein concentration, 4 mL of the chromatographic Ⅶ peak solution (30 mg/mL) was loaded onto a sephadex G-50 gel filtration chromatography column (1.6 × 40 cm) equilibrated with 0.15 mol/L normal saline. Elution was performed with 0.15 mol/L NaCl with a flow rate of 0.5 mL/min at room temperature and monitored at 280 nm. These procedures were performed using an AKTA Purifier. The resulting fractions were collected, desalinated and dehydrated, lyophilized after measuring the concentration, and stored in sealed bottles at −80℃ until needed. 

The NNAV-Ⅶ-2 protein as the target protein was examined using SDS-PAGE, i.e., separating gel (Table 1) and stacking gel (Table 2). The protein samples were mixed with loading buffer and boiled for five minutes. Subsequently, the gels were stained with Coomassie brilliant blue. After decolorization with acetic acid, the protein bands were imaged using a gel imaging system (BIO-PRO). 

**Table 1.** Preparation of separating gel.

Reagent	Volume (µL)
H_2_O	1900
30% Acr-bis	5000
1 mol/LTris (pH 8.8)	2500
10% SDS	100
10% AP	100
TEMED	4

**Table 2.** Preparation of stacking gel.

Reagent	Volume (µL)
H_2_O	2900
30% Acr-bis	830
1 mol/LTris (pH 8.8)	630
10% SDS	50
10% AP	50
TEMED	5

### Identification of target protein

After reduction and alkylation of the above target protein, we subjected the target protein to enzymatic digestion by adding trypsin (mass ratio 1:50) and incubating it for 20 hours at 37℃. Subsequently, the enzyme digestion products were redissolved in 0.1% FA solution and stored at −20℃ for later use after desalting and lyophilizing. Solution A was an aqueous solution of 0.1% formic acid, and solution B was an aqueous acetonitrile solution of 0.1% formic acid (84% acetonitrile). The samples were loaded onto the Trap column by an autosampler after the chromatographic column was equilibrated with 95% liquid A. Peptide mass-to-charge ratios and peptide fragments were collected as follows: 20 fragmentation profiles were collected after each full scan (MS2 scan). Mass spectrometry test raw files were searched against the corresponding databases using Mascot 2.2 software.

### Determination of the analgesic effect of NNAV-Ⅶ-2 using hot plate methods 

The mice were placed on a thermostatic hot plate that was set to 55 ℃ (± 0.5 ℃). The normal pain threshold of each mouse was assessed using the number of licking the hind foot in five minutes as an indicator of pain response. This assessment was performed once every five minutes, repeated three times, and the resulting average value was calculated. We then selected 30 female mice with normal pain thresholds between 5 and 30 seconds. These mice were then randomly divided into three groups, each comprising 10 mice: control group (intraperitoneal injection of normal saline, 0.2 mL/10g), NNAV-Ⅶ-2 group (intraperitoneal injection of NNAV-Ⅶ-2, 0.3 mg/kg), and Ibuprofen group (intraperitoneal injection of Ibuprofen solution, 80 mg/kg, diluted in saline and solubilized with arginine aid). The pain thresholds of mice in these groups were measured at two and four hours after drug administration.

### Effect of NNAV-Ⅶ-2 protein on ASIC1a expression in rats with inflammatory pain

*Experimental groups*

We randomly divided 40 SPF-grade male SD rats weighing 240-260 g into four groups: the control group, formalin group (method for replicating inflammatory pain models as previously mentioned), PcTx-1 group, and NNAV-Ⅶ-2 group. Rats were administered through intrathecal injection 5.5 hours after injection of formalin. The rats were placed in a prone position and underwent intrathecal injections at the L_5-6_ interspinous space as the puncture point [19]. When rats exhibited tail flick responses and cerebrospinal fluid was observed after withdrawing, the drugs were administered slowly. The drugs used were as follows: 0.1 μg/mL of PcTx-1 (diluted in PBS, 20 μL) for the PcTx-1 group, 0.1 μg /mL NNAV-Ⅶ-2 (diluted in PBS, 20 μL) for the NNAV-Ⅶ-2 group, and an equal amount (20 μL) of PBS for the formalin group. The control group used PBS instead of formalin or drugs. Rats were anesthetized through intraperitoneal injection of 1.5% pentobarbital sodium (2 mL/kg) before drug injection.

*Detection of ASIC1a expression in spinal cord tissue using immunohistochemistry*

Rats were sterilized on the skin with 75% ethanol and decapitated six hours after formalin injection to dissect the lumbosacral spinal cord tissues. Subsequently, the tissues were dehydrated and paraffin-embedded. The paraffin blocks were sectioned into slices (4 μm of thickness) and collected onto slides precoated with 0.1% polylysine. The slides were dried at 65 ℃ in an oven, dewaxed and dehydrated, incubated with 3% peroxide for 10 minutes, antigen-retrieved at high temperatures, and naturally dried to room temperature. The sections were then blocked with 10% goat serum for 20 minutes. Subsequently, the sections were incubated with the rabbit anti-rat ASIC1a antibody (1:100) at 4 ℃ for eight hours, followed by incubation with goat anti-rabbit IgG (1:2000) at room temperature for 30 minutes. Between each step, the sections were washed with 0.01 mol/L PBS (pH 7.4) for three minutes three times. These sections were stained with a freshly prepared Diaminobenzidine mixture and restained with hematoxylin. Then, the sections went through bluing, conventional gradient alcohol dehydration, and xylene transparency, and were sealed with neutral gum.

*Detection of ASIC1a expression in spinal cord tissue using Western blot*

Rats were sterilized with 75% ethanol on the skin and decapitated six hours after formalin injection, and the lumbosacral spinal cord tissues were dissected and collected. A lysis solution was added to the tissues, and a homogenate was prepared by crunching with a pestle. The homogenate was centrifuged at 12,000 × g, 4 ℃ for 10 minutes. The supernatant was collected, and its concentration was determined using the BCA kit. Then, the loading buffer was added to the supernatant samples, and the mixture was boiled for 10 minutes. After loading, the protein samples were first stacked at 80 V electrophoresis for 20 minutes and then separated at 120 V electrophoresis for 60 minutes. Subsequently, the protein bands on the gel were transferred to a polyvinylidene fluoride (0.45 μm PVDF) membrane, which was incubated in 5% skimmed milk for blocking at room temperature for two hours. Afterward, the membrane was incubated overnight at 4 ℃ with the rabbit anti-rat ASIC1a antibody (1:2000). The next day, the membrane was washed with TBST three times, 15 minutes each time. Then the membrane was incubated with a goat anti-rabbit secondary antibody (1:8000) at 4 ℃ for 90 minutes with gentle shaking. The membrane was washed with TBST three times, 15 minutes each time. The membrane was incubated with an electrochemiluminescence solution in a dark room, and the bands were recorded using a chemiluminescence gel imaging system (BIO-PRO). 

### Statistical analysis

All data were analyzed using SPSS 16.0 software (IBM Corp., Armonk, NY, USA). The results are presented as means ± SD. Differences were determined using one-way analysis of variance (ANOVA) and bidirectional ANOVA, and *p*-values < 0.05 were considered to be statistically significant.

## Results

###  Isolation of *Naja naja atra* venom 

**Figure 1.**
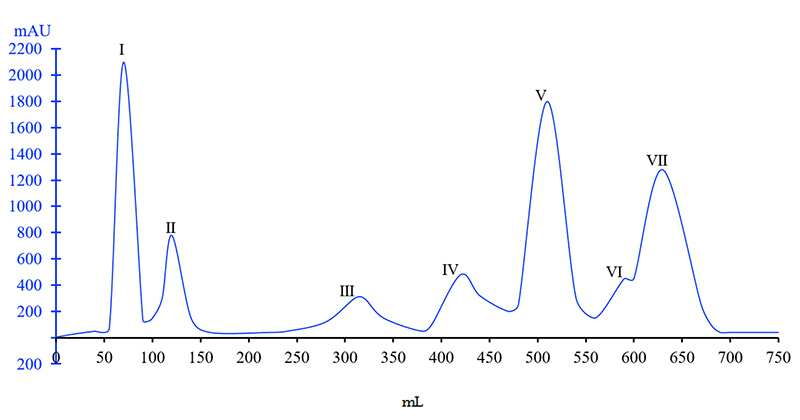
Results of CM Sephadex C-25 column chromatography of venom from *Naja naja atra*. The fractions were eluted with a liner gradient of 0.01 mol/L phosphate buffer (pH 6.0) plus 0.5 mol/L NaCl (400 mL of each of two solutions) at a flow rate of 0.8 mL/min.

After centrifugation and filtration, crude *Naja naja atra* venom was subjected to chromatography on a CM Sephadex C-25 column. The elution of absorbed proteins using a linear gradient of PBS led to seven peaks (Figure 1) and all the eluents were collected. Based on preliminary laboratory research [20] indicating analgesic effects, component Ⅶ was selected for further research. The yield rate of Ⅶ peak was approximately 28%. 

### Capillary electrophoresis to analyze venom protein interacting with ASIC1a

*Capillary electrophoresis of Ⅶ peak samples*

**Figure 2.**
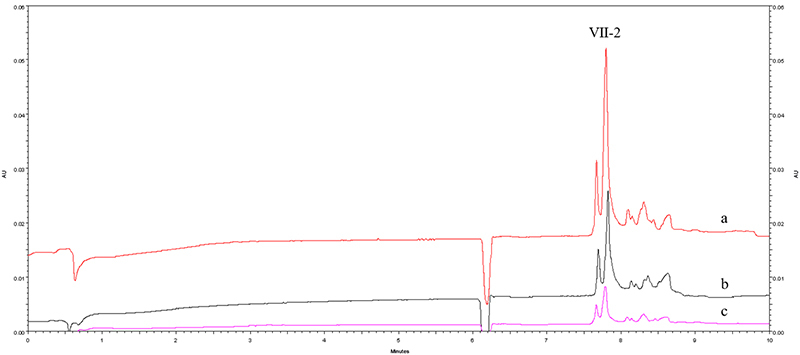
Electropherograms of component Ⅶ with different concentrations. **(a)** 0.6 mg/mL component Ⅶ; **(b)** 0.3 mg/mL component Ⅶ; **(c)** 0.15 mg/mL component Ⅶ. 48.5 cm × 50 μm i.d., 13 kV, 198 nm, 50 mmol/L borate buffer (pH 12.0) as the running buffer. 20 ℃, 3447.38 Pa of the pressure injection for five seconds. All procedures were performed using capillary electrophoresis instrumentation from Beckman Coulter.

**Figure 3.**
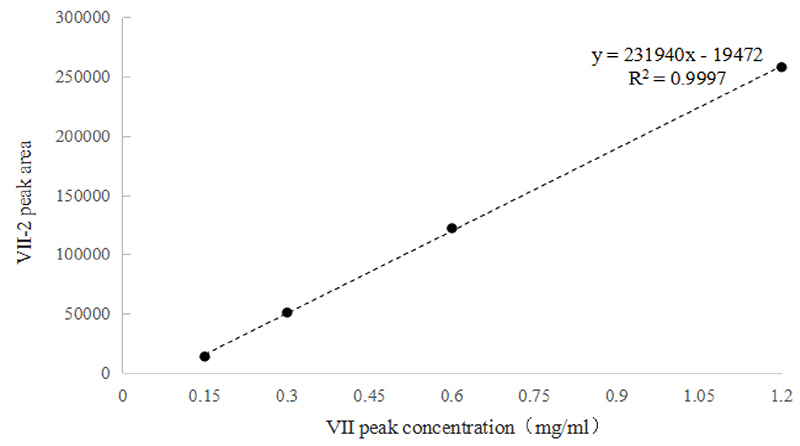
The relationship between the peak area of Ⅶ-2 peak and the mass concentration of Ⅶ peak.

Figure 2 illustrates the capillary electrophoresis results of Ⅶ peak samples with three different concentrations. Component Ⅶ underwent further separation into several peaks under a longer migration time. Notably, the primary peak among them is the second peak (Ⅶ-2), with a migration time of 7.75 minutes and a relative standard deviation (RSD) of 0.83%. Furthermore, the peak area of Ⅶ-2 peak at different concentration demonstrated a good linear relationship with the mass concentration of Ⅶ peak, as shown by the linear equation: y = 231940x - 19472, with a correlation coefficient of R^2^ = 0.9997, as illustrated in Figure 3.

*Capillary electrophoresis of Ⅶ peak-treated samples*

**Figure 4.**
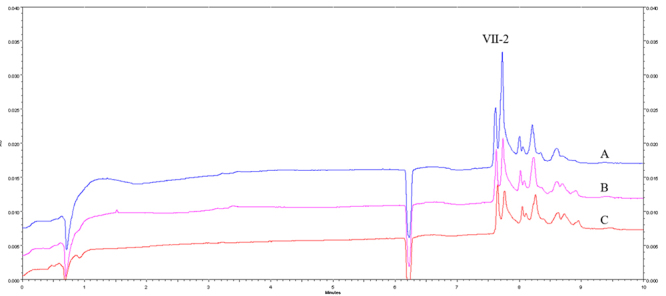
Electropherograms of Ⅶ peak-treated samples incubated at different times. **(A)** Samples incubated for 20 minutes. **(B)** Samples incubated for 10 minutes. **(C)** Samples incubated for five minutes. The electrophoresis conditions were the same as those in Figure 2.

Under the same conditions mentioned above, Figure 4 illustrates the capillary electrophoresis results of Ⅶ peak-treated samples. Compared with the peak area of the untreated sample, the peak area of Ⅶ-2 peak from the treated samples exhibited a reduction of approximately 62.3% after incubation for five minutes, followed by a decrease of 42.8% after incubation for 10 minutes, with no significant difference after incubation for 20 minutes. No significant change was observed in the peak area of other peaks (except Ⅶ-2 peak) in the treated samples. The combination of Ⅶ-2 peak protein and ASIC1a in the plate leads to a decrease in the content of Ⅶ-2 peak protein in the supernatant sample. These findings indicate that the protein could bind to ASIC1a, which we named *Naja naja atra* venom-Ⅶ-2 (NNAV-Ⅶ-2).

### Further purification and the relative molecular mass determination of NNAV-Ⅶ-2 protein 

**Figure 5.**
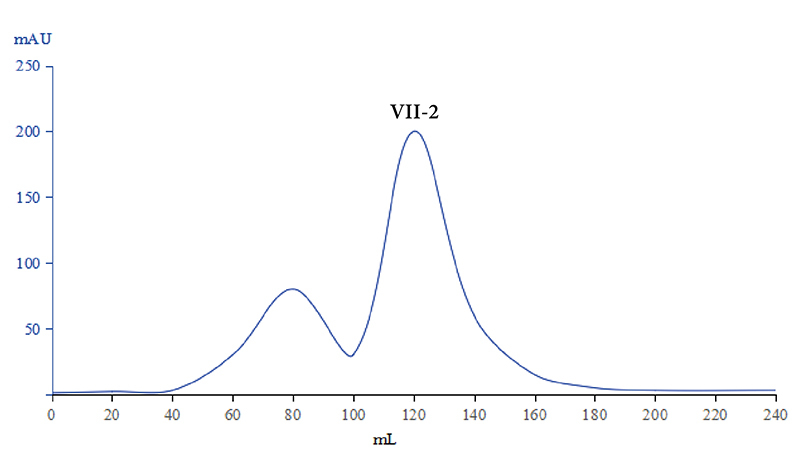
Results of Sephadex G-50 gel filtration chromatography of Ⅶ peak protein. The fractions were eluted with 0.15 mol/L normal saline at a flow rate of 0.5 mL/min.

**Figure 6.**
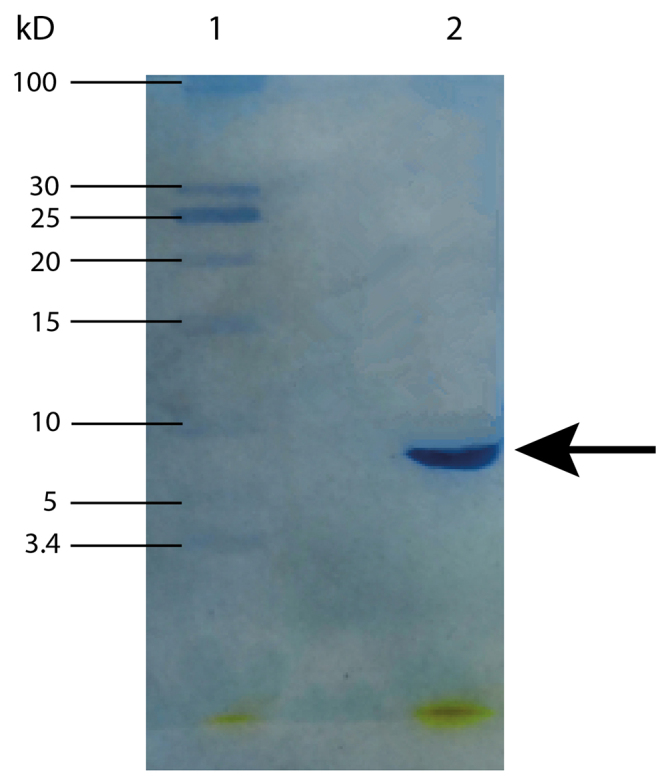
SDS-PAGE results of NNAV-Ⅶ-2. Lane 1: marker; lane 2: NNAV-Ⅶ-2. The arrow indicates the target protein. The protein samples were examined using 5.5% stacking gel and 15.6% separating gel. The gels were stained with Coomassie brilliant blue.

The solution of Ⅶ peak protein using Sephadex G-50 gel filtration led to two peaks (Figure 5). The primary peak (NNAV-Ⅶ-2) is the target protein corresponding to the capillary electrophoresis results. Figure 6 illustrates the SDS-PAGE results of NNAV-Ⅶ-2 protein, which indicated a single band with a relative molecular mass of approximately 6.7 kD.

### Identification of NNAV-Ⅶ-2 protein

**Figure 7.**
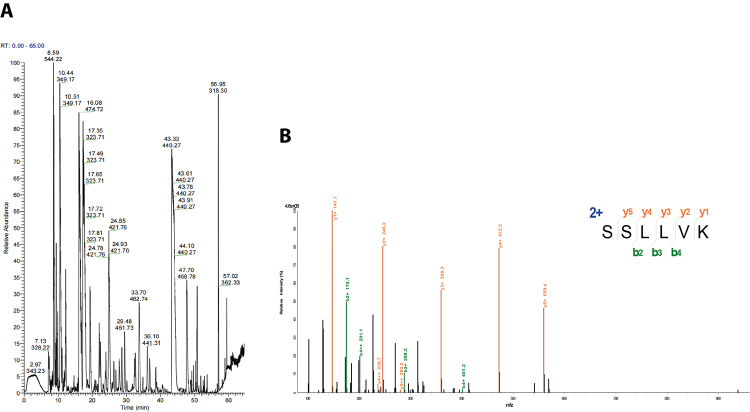
(A) MALDL-TOF/TOF spectra of trypsin peptides from NNAV-Ⅶ-2. (B) MS/MS spectrum of peptide from NNAV-Ⅶ-2.

From the first-order mass spectrometry, we selected the peptide segments with the highest intensities for tandem mass spectrometry analysis. The MS/MS spectra were searched in the NCBInr database using the Mascot search engine; the results are illustrated in Figure 7A. NNAV-Ⅶ-2 protein was identified using a Mascot search MS-BLAST, as shown in Table 3 and Table 4. A total of 17 different peptides were identified with 96.67% cover percent matched with cytotoxin 3, while 14 different peptides were detected with 88.33% cover percent matched with cytotoxin D1. Figure 7B shows the results of the MS/MS spectrum of the target protein with its matching peptide, depicting cytotoxin 3 protein peptide sequence K.SSLLVK.Y with a score of 57.95.

**Table 3.** Identification of NNAV-Ⅶ-2 by MS-BLAST.

Reference	PepCount	UniquePepCount	CoverPercent	MV/PI
pdb|Cytotoxin 3|B Chain B, Cytotoxin 3	457	17	96.67%	6747.25/9.38
prf||1007132A Cytotoxin D1	351	14	88.33%	6810.33/9.38

**Table 4.** Detection of peptides from NNAV-Ⅶ-2 by MS-BLAST and Mascot searches.

Reference	Peptide sequence matched (N-C)	Score
pdb|Cytotoxin 3|B Chain B, Cytotoxin 3	K.LVPLFYK.T	48.09
	K.LVPLFYKTCPAGK.N	56.54
	K.M*FM*VATPK.V	45.83
	K.MFM*VATPK.V	48.77
	K.MFMVATPK.V	48.42
	K.NLCYK.M ! K.NLCYK.I	28.86
	K.NLCYKM*FMVATPK.V	38.19
	K.RGCIDVCPK.N ! K.RGCIDVCPK.S	43.82
	K.SSLLVK.Y	57.95
	K.SSLLVKYVCCNTDR.C	60.07
	K.TCPAGK.N	37.84
	K.TCPAGKNLCYK.M	45.38
	K.YVCCNTDR.C	41.61
	K.YVCCNTDRCN.-	58.71
	R.GCIDVCPK.N ! R.GCIDVCPK.S	57.8
	R.GCIDVCPKSSLLVK.Y	26.13
	K.CNKLVPLFYK.T	24.29

### Analgesic effects of NNAV-Ⅶ-2 protein 

**Figure 8.**
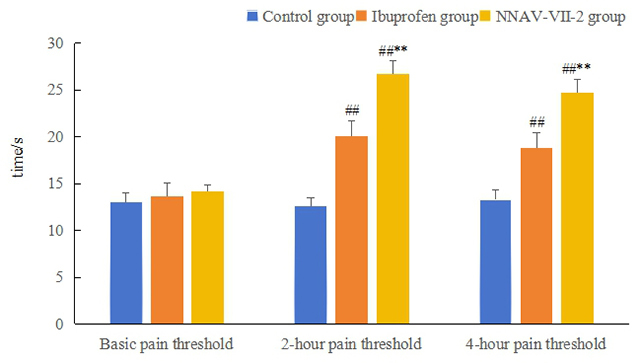
Determination of thermal pain threshold of mice in each group by hot plate method ( $\overset{-}{x}\pm S$ , n = 10). Control group: intraperitoneal injection of normal saline (0.2 mL/10 g). NNAV-Ⅶ-2 group: intraperitoneal injection of an equal amount of NNAV-Ⅶ-2 (0.3 mg/kg). Ibuprofen group: intraperitoneal injection of an equal amount of Ibuprofen solution (80 mg/kg). ^##^ *p* < 0.01 *vs* Control group at corresponding time; ^**^ *p* < 0.01 *vs* Ibuprofen group at corresponding time.

Figure 8 shows the results of the hot plate experiment. The pain thresholds of mice in the Ibuprofen and NNAV-Ⅶ-2 groups were significantly higher two and four hours after the drug administration compared with the control group (*p* < 0.01). Furthermore, the pain thresholds of mice in the NNAV-Ⅶ-2 groups two and four hours after drug administration were significantly higher than those in the Ibuprofen group at the same time (*p* < 0.01).

### Effect of NNAV-Ⅶ-2 protein on ASIC1a expression in rats with inflammatory pain

*Detection of ASIC1a expression in spinal cord tissue using immunohistochemistry*

**Figure 9.**
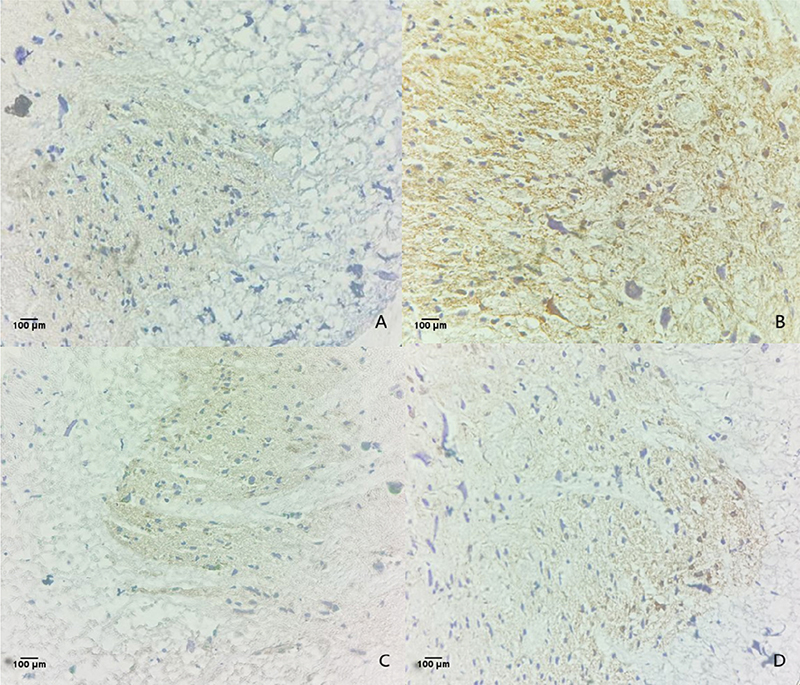
Expression of ASIC1a in spinal cord tissue of rats (×100). **(A)** Control group; **(B)** formalin group; **(C)** PcTx-1 group; **(D)** NNAV-Ⅶ-2 group.

Figure 9 illustrates the immunohistochemical results of ASIC1a expression change in spinal cord tissue. ASIC1a expression in spinal cord tissues was significantly higher in the formalin group than that in the control group. Compared with the formalin group, ASIC1a expressions were significantly reduced in the NNAV-Ⅶ-2 and PcTx-1 groups.

*Detection of ASIC1a expression in spinal cord tissue using Western blot*

**Figure 10.**
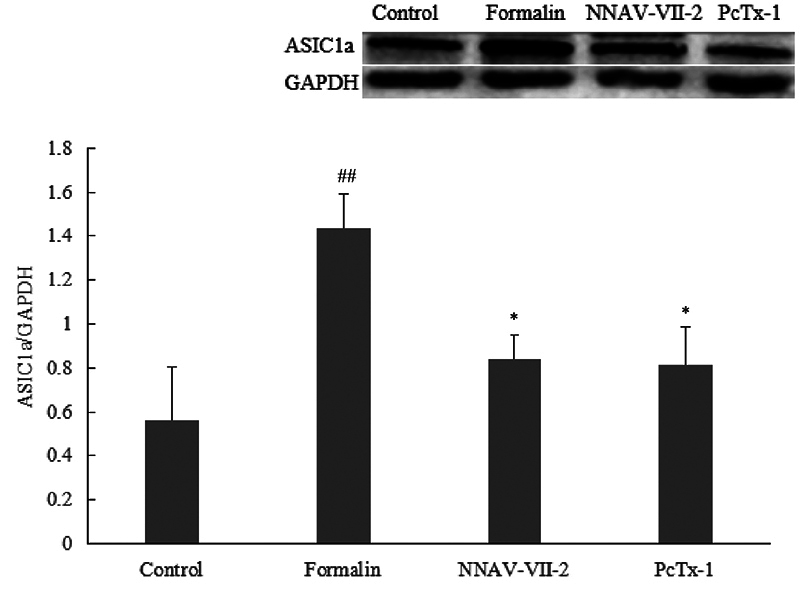
Expression of ASIC1a in spinal cord tissue protein of rats. Formalin group: Intrathecal injection of PBS (20 μL) 5.5 hours after subcutaneous injection of 2.5% formalin solution (100 μL). NNAV-Ⅶ-2 group: Intrathecal injection of NNAV-Ⅶ-2 (diluted in PBS, 0.1 μg/mL) 5.5 hours after subcutaneous injection of 2.5% formalin solution (100 μL). PcTx-1 group: Intrathecal injection of PcTx-1 (diluted in PBS, 0.1 μg/mL) 5.5 hours after subcutaneous injection of 2.5% formalin solution (100 μL). Control group: Intrathecal injection of PBS 5.5 hours after subcutaneous injection of PBS (100 μL). ^##^ *p* < 0.01 *vs* Control group; ^*^ *p* < 0.05 *vs* formalin group.

Figure 10 illustrates the western blot results. ASIC1a expression in the dorsal horn of the spinal cord was significantly elevated in the formalin group compared with the control group (*p* < 0.01). Conversely, compared with the formalin group, both the NNAV-Ⅶ-2 group (*p* < 0.05) and the PcTx-1 group (*p* < 0.05) exhibited a considerable reduction in ASIC1a expression in the dorsal horn of the spinal cord, with no statistical difference between the two groups.

## Discussion

In various pathological conditions, such as pain [21], ischemic injury [22], tumor [23]*,* and epilepsy [24], distinct characteristics, including acidification of the local tissue environment and a decrease in pH, were observed. This environmental acidification causes a significant activation of ASICs, leading to corresponding physiological and pathological reactions, stimulating the secretion of inflammatory factors, exacerbating vascular endothelium, and promoting tumor growth and metastasis. To further clarify the biological function of ASICs and their relationships with disease occurrence and development, investigating and developing more ASICs regulators is a crucial breakthrough to addressing this challenge. Currently, researches on ASICs regulators primarily involve patch-clamp experiments [25-27]. Due to the low flux of this conventional patch clamp method, the operation process is complex and cumbersome, and parallel detection cannot be conducted. Additionally, the whole-cell patch clamp method is only suitable for suspension cell experiments, and the consumables are expensive. Thus, it is particularly crucial to develop a simple and effective detection approach.

Our laboratory has confirmed the presence of nonaddictive analgesic components in the *Naja naja atra* venom [20]. Nonetheless, whether these components exert analgesic effects through interaction with ASIC1a remains unexplored. In this study, cation exchange chromatography was employed to isolate seven chromatographic peak components from *Naja naja atra* venom. Previous investigations have indicated the analgesic effects of the peak Ⅶ component [28, 29]. Consequently, component Ⅶ was selected as a sample for analysis. We established a capillary electrophoresis method to detect the component Ⅶ with various concentrations. The findings show that the peak area of the primary peak (Ⅶ-2) with RSD of 0.83% exhibited a good linear relationship with the mass concentration of Ⅶ peak. These demonstrate the high repeatability and stability of the capillary electrophoresis approach under the above experimental conditions. 

The preliminary experimental results showed that component Ⅶ did not bind to the coupled anti-rat ASIC1a antibody microplate (uncoupled ASIC1a) after incubation at different times. Cell membrane ASIC1a protein is essential for the experiment to detect whether component Ⅶ contains proteins that can interact with ASIC1a. Our previous investigation revealed that the expression of ASIC1a in the spinal cord tissue of rats was the most significant at six hours after formalin injection, consistent with the findings reported by Zu et al. [30]. In this study, the extracted protein from spinal cord tissue was coupled to the bottom of an enzyme-linked immunosorbent assay plate containing anti-rat ASIC1a antibody, then a specific concentration of component Ⅶ was introduced to the bottom of the microplate hole and incubated for varying durations at 37 ℃. Subsequently, the supernatant from the microplate hole was collected, and capillary electrophoresis was performed under the same conditions as indicated above. The change in content was analyzed based on the change in peak area of the Ⅶ-2 peak. The findings demonstrate that the binding of Ⅶ-2 to ASIC1a reaches its maximum at five minutes of incubation, with no significant difference after incubation for 20 minutes compared with the untreated Ⅶ-2 peak samples at the same concentration. No significant change was observed in the peak areas of other peaks (except Ⅶ-2). These findings strongly indicate the interaction of the Ⅶ-2 protein with ASIC1a, and the combination and dissociation between them are fast. Thus, this method based on capillary electrophoresis offers a straightforward and sensitive approach for detecting and analyzing venom components interacting with ASIC1a.

The protein of Ⅶ-2 peak was obtained through gel filtration and was analyzed using SDS-PAGE. This analysis exhibited a single band with a relative molecular mass of approximately 6.7 kD, which was similar to the previous findings reported by Yu Ling in our laboratory [20]*.* However, there was insufficient research on the identification and analgesic activity of protein Ⅶ-2*.* In this study, the findings from mass spectrometry analysis indicated that 17 different peptides were detected, with a peptide coverage of 96.67% paired with cytotoxin 3. It has been previously reported that [31] cytotoxin 3 derived from Cobra venom also exhibits analgesic effects, but its relationship with ASICs has not been explored.

Considering the extreme sensitivity of the testicles of male mice to heat stress, which affects the observation of foot licking, female mice (in diestrus) were selected for the hot plate test. The results revealed that the analgesic effect of NNAV-Ⅶ-2 protein was more substantial and lasted longer than that of Ibuprofen. PcTx-1 with a molecular weight of 4.7 kD is a toxin that could bind to the subunit interface of ASIC1a. The expression of ASIC1a in the spinal cord tissue of rats with inflammatory pain was considerably reduced 30 minutes after the intrathecal injection of PcTx-1. NNAV-Ⅶ-2 with the same mass concentration as PcTx-1 could also cause a significant downregulation of ASIC1a, and there is no statistically significant difference between them. We speculate that the analgesic mechanism of the NNAV-Ⅶ-2 may be associated with its binding to ASIC1a, resulting in the downregulation of ASIC1a expression in neural tissues. Further research is needed regarding the mechanism by which PcTx-1 and NNAV-Ⅶ-2 cause ASIC1a downregulation.

## Conclusion

In conclusion, this method based on capillary electrophoresis offers a straightforward and sensitive approach for detecting and analyzing venom components interacting with ASIC1a. The analgesic mechanism of the NNAV-Ⅶ-2 protein may be associated with its binding to ASIC1a, resulting in the downregulation of ASIC1a expression in neural tissues. These results offer a crucial theoretical foundation for further investigating ASICs functions and the analgesia mechanisms of toxins.

## Data Availability

The datasets generated and analyzed during the current study are available from the corresponding author upon reasonable request.
